# 715. Dynamic gain and loss of carbapenem-resistant Enterobacterales by VHA patients during 2016-2022: loss of CRE exceeds gain of CRE

**DOI:** 10.1093/ofid/ofad500.777

**Published:** 2023-11-27

**Authors:** Andrew Chou, David J Ramsey, Barbara Trautner

**Affiliations:** Michael E. DeBakey VA Medical Center, Baylor College of Medicine, Houston, Texas; Michael E. DeBakey Veterans Affairs Medical Center, Houston, Texas; Michael E. DeBakey Veterans Affairs Medical Center / Baylor College of Medicine, Houston, TX

## Abstract

**Background:**

Carbapenem-resistant Enterobacterales (CRE) are an important public health threat. The long-term prognosis and duration that patients harbor CRE is not well described.

**Methods:**

We conducted a retrospective study of patients who had a culture that grew CRE within the VHA from 2016-2022. For these subjects, all microbiology data was extracted from the VHA database. CRE was defined using the VHA 2019 definition: *Escherichia coli*, *Klebsiella*, *Enterobacter*, or *Citrobacter* spp. & resistant to imipenem, meropenem, or doripenem. The first CRE isolate was considered the index CRE culture, and its collection date was set as time 0 (Figure 1). Cultures within 30 days of the index CRE culture were not included in the analysis. Cultures were assigned one to the following time periods: 6 months, 1 year, 2 years, 3 years, 4 years, and after 4 years. Time periods when culture(s) grew CRE were labelled ‘R’, time periods when cultures grew bacteria that were not CRE were labelled ‘S’, time periods when no cultures were collected were labelled ‘N’, time periods during and after patient’s death were labelled ‘D’.

**Figure 1:**
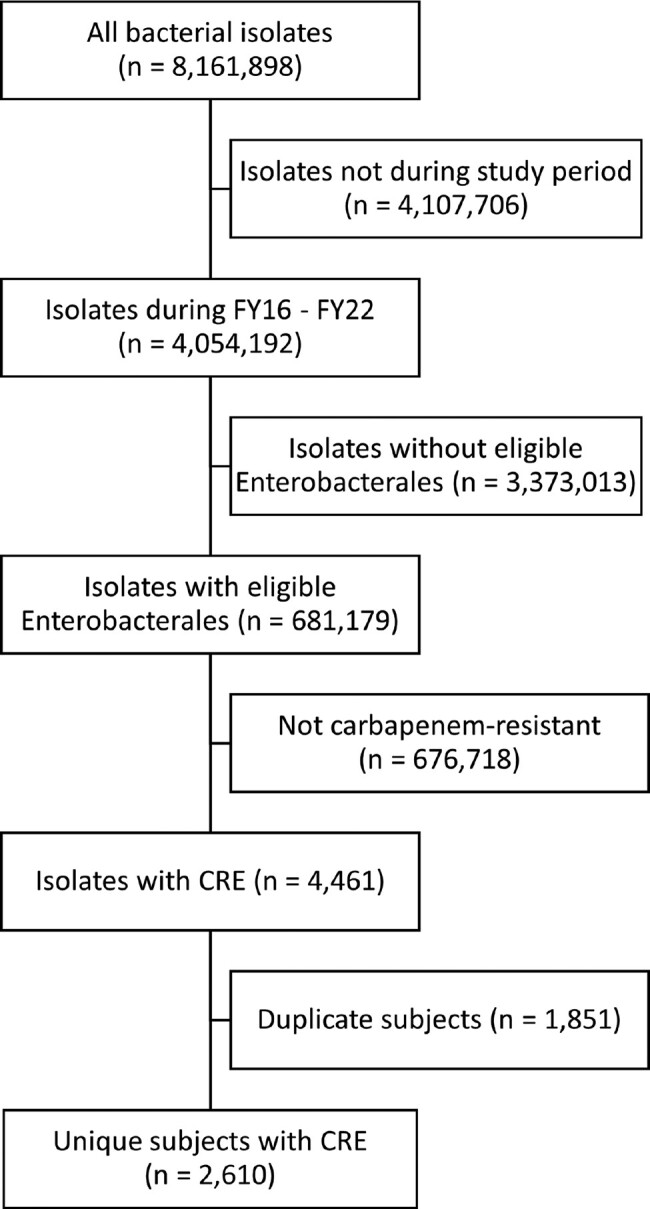
Identification of eligibility

**Results:**

We identified 8,161,898 isolates during the study period, of which 681,179 had eligible Enterobacterales. There were 4,461 CRE isolates from 2,609 unique patients. The Sankey diagram (Figure 2) shows the dynamics after the index CRE culture. At 6 months after index CRE culture, 15% of subjects still had a CRE+ culture. At 1 year and 2 years, 8% subjects still had CRE. Importantly, during each time period, study subjects steadily changed from R (grew CRE during time period) to S (grew non-CRE during time period). The rate of change from R to S remained steady throughout all time periods (range: 24% - 36%) (Table 2). The rate of re-gaining CRE (ie. status change from S to R, N to R) was low (range: 0% to 6%). The 4-year survival after the index CRE culture was 45%.

**Figure 2:**
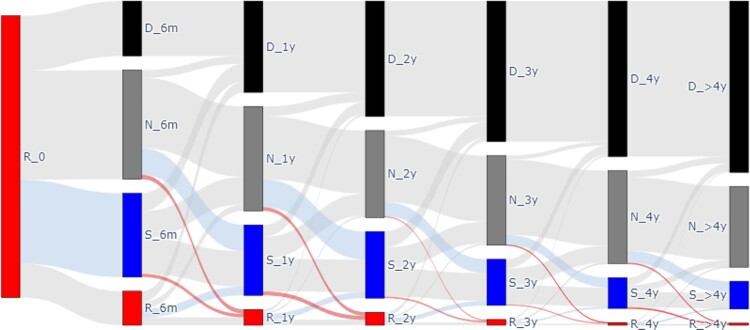
Sankey diagram shows dynamic CRE status changes over time

R status (red nodes): CRE grew during the prior time period. N status (grey nodes): no microbiology cultures collected during prior time period. S status (blue nodes): non-CRE grew during the prior time period; D status (black nodes): patient died. Red ribbons: indicates conversion from N status to R status, or S status to R status; blue ribbons: indicates conversion from R status to S status, or N status to S status. Nodes are vertical bars at each time period (6 months, 1 year, 2 years, 3 years, 4 years, and >4 years after index CRE+ culture. Links are ribbons between nodes.

**Table 1:**
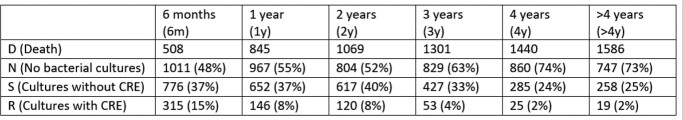
Risk for CRE+ culture after index CRE culture (time 0)

**Table 2:**
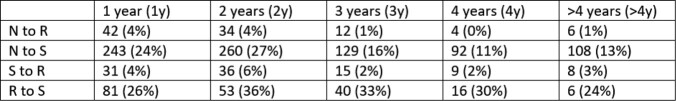
CRE+ culture gain & loss dynamics over time

N: no bacterial cultures; S: cultures without CRE; R: cultures with CRE. Percentages are the number of subjects that changed status divided by total at prior state. For example N to R: 42 (4%) means 42 subjects changed from N to R status during the time period. 4% represented the 42 subjects that changed status were out of 1,011 that started the time period in N status (42/1011 = 4%).

**Conclusion:**

After an index CRE culture, subjects steadily changed from R status (grew CRE during time period) to S status (grew non-CRE during time period) at the rate of 30% to 36% per year. After subjects converted to S status (grew non-CRE during time period), the rate of reversion to R status (grew CRE during time period) was 2% to 6% per year.

**Disclosures:**

**Andrew Chou, MD, MSc**, Entasis Therapeutics: Stocks/Bonds **Barbara Trautner, MD, PhD**, Genentech: Grant/Research Support|Peptilogics: Grant/Research Support

